# Protein stickiness, rather than number of functional protein-protein interactions, predicts expression noise and plasticity in yeast

**DOI:** 10.1186/1752-0509-6-128

**Published:** 2012-09-27

**Authors:** Leandra M Brettner, Joanna Masel

**Affiliations:** 1 Present address: Ecology & Evolutionary Biology, University of Arizona, 1041 E Lowell St, Tucson, AZ, 85721, USA; 2 Present address: Bioengineering, University of Washington, 3720 15th Ave NE, Seattle, WA, 98195, USA

**Keywords:** Protein-protein interaction networks, Stochastic gene expression, Evolutionary constraint, Correlomics, Cooperativity, Phenotypic plasticity

## Abstract

**Background:**

A hub protein is one that interacts with many functional partners. The annotation of hub proteins, or more generally the protein-protein interaction “degree” of each gene, requires quality genome-wide data. Data obtained using yeast two-hybrid methods contain many false positive interactions between proteins that rarely encounter each other in living cells, and such data have fallen out of favor.

**Results:**

We find that protein “stickiness”, measured as network degree in ostensibly low quality yeast two-hybrid data, is a more predictive genomic metric than the number of functional protein-protein interactions, as assessed by supposedly higher quality high throughput affinity capture mass spectrometry data. In the yeast *Saccharomyces cerevisiae*, a protein’s high stickiness, but not its high number of functional interactions, predicts low stochastic noise in gene expression, low plasticity of gene expression across different environments, and high probability of forming a homo-oligomer. Our results are robust to a multiple regression analysis correcting for other known predictors including protein abundance, presence of a TATA box and whether a gene is essential. Once the higher stickiness of homo-oligomers is controlled for, we find that homo-oligomers have noisier and more plastic gene expression than other proteins, consistent with a role for homo-oligomerization in mediating robustness.

**Conclusions:**

Our work validates use of the number of yeast two-hybrid interactions as a metric for protein stickiness. Sticky proteins exhibit low stochastic noise in gene expression, and low plasticity in expression across different environments.

## Background

A protein that functionally interacts with many other proteins may be more sensitive to noise in gene expression [1]. In agreement with this prediction, a negative correlation between noise and protein-protein interaction (PPI) degree has been found [2,3]. However, PPI datasets are notorious for high rates of false positive and false negative interactions [4-7]. Older high throughput datasets rely on yeast two-hybrid (Y2H) studies, which can measure interactions between two proteins that would never even encounter each other in nature. More recently, high throughput affinity capture mass spectrometry (ACMS) data have become available [8], which do not suffer from this drawback and subsequent high false positive rate.

**Figure 1:**
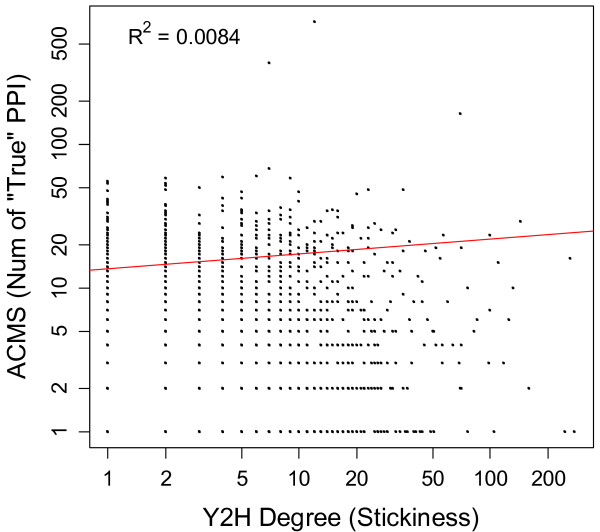
**A protein’s number of PPIs correlates poorly across two high throughput data types.** Model 1 regression line is shown for illustrative purposes only, to show the weakness of the correlation.

Y2H data may indicate the non-specific “stickiness” of a protein towards a random polypeptide better than it indicates the number of functional protein-protein interactions that the protein is involved in [9,10]. The numbers of PPIs per protein (node degrees) for Y2H vs. ACMS data are only weakly correlated in yeast (Figure 1, R^2^ = 0.008, p = 2e-05). Y2H and ACMS data clearly measure different things.

Here we find that Y2H degree/stickiness predicts gene noise better than ACMS/“real PPI” degree does. It has previously been argued that a protein that functionally interacts with many other proteins will be more sensitive to noise [1]. However, if a protein binds promiscuously to many non-functional partners, variable expression of that protein may be extremely disruptive to diverse processes, also leading to a negative correlation, in this case between the number of false positive PPIs and noise. By itself, ACMS degree is correlated with noise, perhaps due to residual sticky but non-functional false positives in the ACMS data. But ACMS drops out as a statistically significant predictor in a multiple regression when Y2H degree, a better estimator of non-specific protein-protein binding, is included.

Here we also introduce a new metric of plasticity, i.e. the variation in gene expression across different experiments. Again, we find that Y2H degree/stickiness predicts plasticity better than higher quality ACMS data on the number of functional PPIs. For both noise and plasticity, our results are robust to a multiple regression analysis that controls for protein abundance and that controls noise for plasticity and vice versa. Other significant predictors include the presence of a TATA box, whether a protein forms a homo-oligomer, and gene essentiality.

## Results

**Figure 2:**
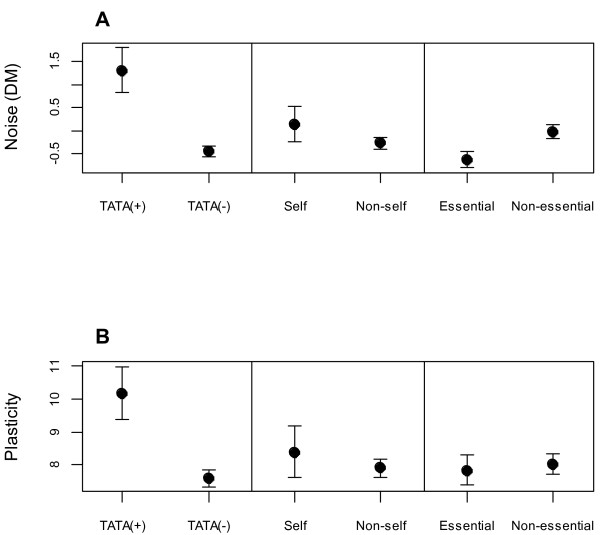
**Illustration of binary predictors of noise and plasticity, taken in isolation.** The presence of a TATA box strongly predicts noise and plasticity. Homo-oligomerization does not, in isolation, predict plasticity, and its effect on noise is only marginally statistically significant (p = 0.0496). However, these effects become significant when confounding factors are accounted for (Tables 1 and 2). Essentiality predicts noise but not plasticity. To better assess effect sizes using more intuitive noise and plasticity measures, back transformations were performed to restore original units. The mean plasticity residual was added to the mean Box-Cox transformed plasticity score, and then the Box-Cox transform was reversed, so that plasticity corresponds simply to the estimated number of experiments for which expression varies. The noise axis corresponds to the DM metric of Newman et al. [13]. Error bars correspond to 95% confidence intervals.

Table 1 summarizes the results of regression analyses predicting noise. After extensive bottom-up and top-down multiple regression model building, the best model (first numeric column: total coefficient of determination R^2^ = 0.1083) included Y2H, but not ACMS, as a predictor of noise. The second numeric column shows the R^2^ when a single predictor is considered in isolation. These values are given as a contrast to our primary results in the first numeric column, which show the extent to which the coefficient of determination is reduced if a predictor is removed from the best model. For the purposes of more graphical illustration, Figure 2A also shows the effect of each binary predictor in isolation.

**Table 1:** Multiple regression results predicting noise

**Factor**		**Subtract Factor from Best Model Predicting Noise**	**Factor in Isolation**	**Subtract Factor from Best Model Predicting Noise**	**Factor in Isolation**
				** Plasticity Included in Model**	
**Y2H PPI**	R^2^	0.0093	0.0100	0.0064	0.0121
	p	***	***	**	***
**ACMS PPI**	R^2^	ns	0.0081	ns	0.0114
	p	-	**	-	***
**TATA Box (+/−)**	R^2^	0.07555^1^	0.0779	0.0602^2^	0.0752
	p	***	***	***	***
**Self Interaction (+/−)**	R^2^	0.0067	0.0033	0.0038	0.0045
	p	**	*	*	*
**Gene Essentiality (+/−)**	R^2^	0.01744^1^	0.0169	0.0161^1^	0.0211
	p	***	***	***	***
**Plasticity**	R^2^	-	-	0.0495^3^	0.0781
	p	-	-	***	***
**TATA × Essentiality**	R^2^	0.0098	-	0.0060	-
	p	***	-	**	-
**Plasticity if TATA(+)**	R^2^	-	-	0.0279	0.0815
	slope	-	-	0.0010	0.0015
	p	-	-	***	***
**Plasticity if TATA(−)**	R^2^	-	-	0.0219	0.0281
	slope	-	-	0.0004	0.0005
	p	-	-	***	***
**TATA × Plasticity**	R^2^	-	-	0.0075	-
	p	-	-	**	-

^1^ also removed TATA × Essentiality.

^2^ also removed TATA × Essentiality, Plasticity if TATA(+), Plasticity if TATA(**−**), and restored Plasticity.

^3^ removed Plasticity if TATA(+) and Plasticity if TATA(**−**).

Models without (1^st^ two numeric columns) and with (last two columns) plasticity as a predictor are shown. After extensive model building, we found that high noise is predicted by low stickiness (low Y2H degree), presence of a TATA box, ability to bind itself, non-essentiality, and high plasticity. A statistically significant interaction term between TATA presence and non-essentiality shows that these two factors have synergistic effects. The TATA × plasticity interaction term is also statistically significant (last row). To provide greater insight, we transformed 3 terms (TATA, plasticity and their interaction) into more intuitive forms (TATA, plasticity if TATA(+), plasticity if TATA(−)). The slope coefficient for plasticity if TATA(+) is 2.5 times larger than that for plasticity if TATA(−), but they make similar contributions to R^2^ due to the much larger number of TATA(−) genes. R^2^ values are shown for each predicting factor in isolation (2^nd^ and 4^th^ numeric columns), as well as, more importantly, for the reduction in the total coefficient of determination R^2^ when the factor is removed from the best model (1^st^ and 3^rd^ numeric columns). Sometimes, as indicated in the footnotes, this involved removing multiple terms and reversing the interaction factor transformation to get a biologically interpretable result. “ns” indicates p > 0.05, * p < 0.05, ** p < 0.01, *** p < 0.001.

The strongest predictor for noise is the presence of a TATA box, consistent with earlier findings: TATA boxes are associated with higher noise [11-13]. Gene essentiality is also an important predictor of gene noise, again consistent with earlier findings that essential genes have lower noise [1,2,13,14]. We also found a statistically significant interaction term, with genes that are both non-essential and possess a TATA box having higher noise than would be expected from the two factors in isolation.

Genes that interact with themselves (form homo-oligomers) have higher noise than genes that do not self-associate. Explanations for this novel finding are explored further at the end of the Results section and in the Discussion.

**Figure 3:**
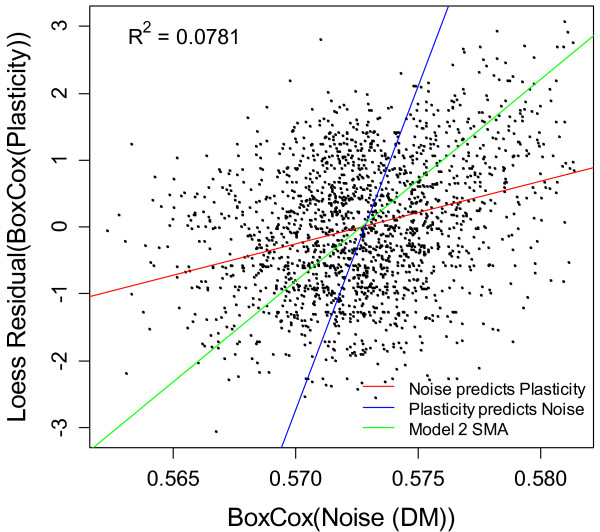
**A gene’s noise and plasticity are correlated.** Multiple regression analyses in Tables 1 and 2 use Model 1 regression, but with reversed dependent and independent variables. For such a weak correlation, plasticity as a function of noise is quite different from the inverse function of noise as a function of plasticity: both lines are shown here. In the absence of a correlation, the functions describing these two lines would be horizontal and vertical, respectively. For comparison, the Model 2 Standard Major Axis regression line is also shown. The correlation between noise and plasticity is tighter in the top right corner, where values of both are high [11].

Genes with high noise in a single environment tend also to have high variation across different environmental conditions (plasticity), due at least in part to mechanistic coupling at the promoter level [2,11,15-18] (Figure 3). In order to infer variables that affect noise reliably, it is therefore important to correct for plasticity. Previous metrics of plasticity have been based on the average pair-wise ratio between microarray spot densities across a variety of environmental conditions [11,12,19,20]. However, the dynamic range of microarray signals depends on transcript abundance [21], making this plasticity metric dependent on abundance. Here we construct a plasticity metric that is less abundance-dependent by design, and which we then correct for residual effects of protein abundance (see Methods). Note that our estimate of protein noise has already been corrected for protein abundance [13]. It is important to correct noise and plasticity for abundance before testing their correlation with PPI degree, since PPI degree can be confounded with abundance [22]. Indeed, protein abundance is an important constraint on evolution, and so may affect a wide range of properties [23].

When we correct for plasticity, our main results on predictors of noise all still hold (Table 1, last two columns, total coefficient of determination R^2^ rises to 0.1609), although many of the R^2^ values attributable to specific predictors are modestly reduced. Correlations between noise and plasticity are known to be stronger in genes containing a TATA box [11]. In agreement with this, the TATA × Plasticity interaction term is statistically significant. Plasticity predicts noise both for TATA(+) genes and for TATA(−) genes, but the effect size (i.e. regression coefficient or slope) is 2.5 times as large for TATA(+) genes (Table 1).

Next, we considered how PPI metrics and other factors predict plasticity (Table 2, total coefficient of determination R^2^ =0.1267) for that subset of genes for which noise data were also available. Note that this requirement for the availability of noise data biases analyses towards the properties of higher-abundance proteins. Fortuitously, this makes ACMS a more reliable metric of “true” PPIs [5,24], strengthening our interpretation of the results.

**Table 2:** Multiple regression results predicting plasticity

**Factor**		**Subtract Factor from Best Model Predicting Plasticity**	**Factor in Isolation**
**Y2H PPI**	R^2^	0.0191^1^	0.0194
	p	***	***
**ACMS PPI**	R^2^	ns	0.0040
	p	-	*
**TATA Box (+/−)**	R^2^	0.0242^2^	0.0445
	p	***	***
**Self Interaction (+/−)**	R^2^	0.0087^3^	0.0015
	p	**	ns
**Gene Essentiality (+/−)**	R^2^	ns	0.0005
	p	-	ns
**Noise**	R^2^	0.05344^4^	0.0781
	p	***	***
**Noise if TATA(+)**	R^2^	0.0315	0.0449
	slope	167.24	0.9949
	p	***	***
**Noise if TATA(−)**	R^2^	0.0224	0.0438
	slope	70.444	−0.9858
	p	***	***
**TATA × Noise**	R^2^	0.0085	-
	p	**	-
**Y2H if Self**	R^2^	0.0123	0.0004
	slope	−0.3145	−0.0221
	p	***	ns
**Y2H if Non-self**	R^2^	0.0070	0.0154
	slope	−0.0839	−0.1113
	p	**	***
**Y2H × Self Interaction**	R^2^	0.0059	-
	p	**	-

^1^ removed Y2H if Self and Y2H if Non-self.

^2^ also removed Noise if TATA(+), Noise if TATA(**−**) and restored Noise.

^3^ also removed Y2H if Self and Y2H if Non-self and restored Y2H PPI.

^4^ removed Noise if TATA(+) and Noise if TATA(**−**).

After extensive model building, we found that high plasticity is predicted by low stickiness (low Y2H degree), presence of a TATA box, ability to bind itself, and high noise. The TATA × noise and self-interaction × Y2H interaction terms are also statistically significant. To provide greater insight, we transformed the interaction terms as described in the Table 1 legend. R^2^ values are shown for each predicting factor in isolation (last column), as well as, more importantly, for the reduction in the total coefficient of determination R^2^ when the factor is removed from the best model. Sometimes, as indicated in the footnotes, this involved removing multiple terms and reversing the interaction factor transformation to get a biologically interpretable result. “ns” indicates p > 0.05, * p < 0.05, ** p < 0.01, *** p < 0.001.

As with low noise, high Y2H degree/stickiness predicts low plasticity but many ACMS/“functional” PPIs do not. This plasticity correlation holds true even after correcting for the effects of noise. This may be because promiscuous binding poses a particular challenge when it occurs at different extents in different environments. Or it may be because the successful fulfilment of the function of a plastic gene, which requires different levels of expression in different environments, is more sensitive to the effects of a given quantity of noise.

Gene essentiality predicts noise, but it does not predict plasticity. This could be because many genes are only essential in some environments or cell cycle stages, rather than constantly. In agreement with previous findings [11], the presence of a TATA box predicts plasticity as well as noise.

**Figure 4:**
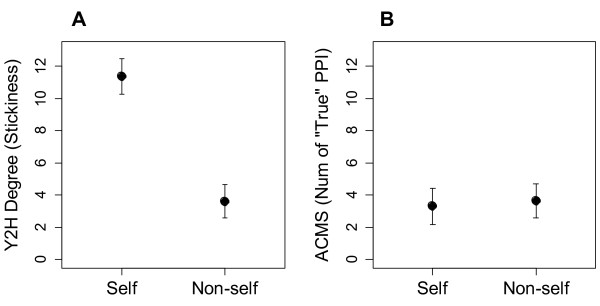
**Proteins that homo-oligomerize are stickier, but do not have more functional PPIs. Analyses were performed on log(PPI) and back-transformed to yield more intuitive PPI metrics. 95% confidence intervals are shown**.

A novel finding of this paper is that the ability to homo-oligomerize predicts both noise and plasticity. Sticky proteins that bind promiscuously are also more likely to stick to themselves [25]. This means that self-interaction and Y2H are both surrogate metrics for intrinsic protein stickiness. In agreement with this interpretation of Y2H, but not ACMS, as a measure of “stickiness”, self-interaction correlates with Y2H but not ACMS (Figure 4).

However, in our multiple regressions, supposedly sticky self-interacting proteins had higher rather than lower noise and plasticity. In the Discussion, we explore possible causes of this relationship. The relationship can only be seen when stickiness is first controlled for, via Y2H data, in a multiple regression analysis. Deceptively, correlations between homo-oligomerization and noise or plasticity were weak to non-existent in single factor analyses (Table 1, Table 2, Figure 2), where stickiness is a confounding factor.

Unsurprisingly given that both correlate with stickiness, the Self-Interaction × Y2H interaction term is statistically significant in our predictive model of plasticity. Y2H predicts plasticity more strongly (i.e. with a larger slope/coefficient) for the already-sticky self-interacting proteins than for non-self-interacting proteins.

## Discussion

Avoiding non-functional PPIs is an important constraint in protein evolution [9,26-28]. Use of the number of Y2H interactions as a validated metric of non-functional PPIs, or “stickiness”, has the promise to reveal more about the nature and consequences of this constraint. Here we have contributed to this validation by showing that Y2H degree is a better predictor of gene expression noise, plasticity, and likelihood of homo-oligomerization than the supposedly superior ACMS data on “true” protein-protein interactions. Given that Y2H data are known to be poor indicators of functional PPIs, our results imply that Y2H data can nevertheless yield a metric with real biological meaning.

We also found that proteins that homo-oligomerize had higher noise and higher plasticity, after confounding factors (including PPI [25]) were controlled for. High variation in protein abundance (noise) does not necessarily correspond linearly with high variation in protein activity. To explain our results, we hypothesize that homo-oligomerization decreases the sensitivity of protein activity to stochastic noise in protein abundance. Plastic genes, which require different levels of activity in different environments, may be more sensitive to the effects of a given quantity of noise, explaining why plasticity is also predicted by homo-oligomerization.

Two very different mechanisms may explain how homo-oligomerization decreases the sensitivity of protein activity to stochastic noise in protein abundance, depending on whether the active form of the protein in question is a monomer or a homo-oligomer. First, consider the case where the monomer is the active form. Homo-oligomerization may act as a sequestration sink that depends in a stronger than linear fashion on concentration. This creates robustness to noise by making the active monomer concentration less dependent on the total level of expression of that protein [29,30]. Sequestration via homo-oligomers rather than hetero-oligomers could help prevent concentration changes from cascading through the PPI network [31].

**Figure 5:**
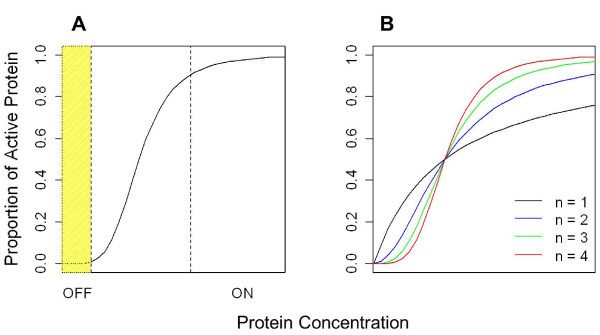
**Sigmoidal dose–response curves of cooperative proteins.A**) In the shaded area, cooperativity suppresses the effects of gene expression noise, preventing inappropriate pathways from being switched on. **B**) Dose–response curves shown for Hill coefficients of 1, 2, 3, and 4.

If the homo-oligomer is the active form, noise in protein abundance can be mitigated by switch-like kinetics (i.e. a sigmoidal dose–response curve) [32]. With a switch, increasing gene expression has a negligible effect until a critical threshold concentration is reached (Figure 5A). The response is then rapidly amplified until near saturation. Sigmoidal kinetics attenuate the effects of noise by allowing the cell to react only to stimuli of an adequate magnitude [32]. Sigmoidal kinetics control noise by controlling the level of activity, rather than by closely regulating the concentration of a signal molecule.

Protein cooperativity is a common example of a switch-like system [33,34]. For example, the active form of a protein may consist of several subunits, each with a binding site. In a cooperative protein, the binding of one subunit to one target will lead to a conformational change that has allosteric effects on the other subunits. This will in turn affect the target binding affinity of the other sites. For example, when oxygen binds to one of the four subunits of hemoglobin, the entire complex relaxes, allowing oxygen to bind more easily to the other three subunits. With each successive binding, oxygen is taken up more readily. The binding activity of hemoglobin increases with the amount of available substrate. This leads to the “all or nothing” switching response. Cooperative systems follow the Hill equation θ = [P]^n^/(K_d_ + [P]^n^) where θ is the proportion of active protein and *n* is the Hill coefficient, which is related to, but often lower than, the number of subunits in the active complex (Figure 5B) [33].

Cooperative binding means that sigmoidal all-or-nothing response curves are likely to be more common for homo-oligomers. This could mean that the evolution of homo-oligomerization is favored for gene products whose activity needs to be robust to accidental activation, for example if gene expression is particularly noisy, or if plasticity is critical, with strong selection against accidently turning on an inappropriate pathway [35]. The *de novo* evolution of active homo-oligomers is likely to be rare, making causality more plausible in the opposite direction, at least under the cooperativity rather than the sequestration explanation of our results. In other words, given a homo-oligomer that reduces the consequences of any expression noise, relaxed selection allows greater noise and plasticity to evolve, either against a background of constant gene function, or in terms of which new functions are likely to evolve within a gene family. Whichever direction causality operates, we expect homo-oligomerization to be correlated with noisy and plastic gene expression, in agreement with our regression analyses. This effect became statistically detectable only after we accounted for the important confounding factor of protein stickiness, which our results suggest can be measured using Y2H data.

## Conclusions

Our work validates use of the number of Y2H interactions as a metric for protein stickiness. Sticky proteins, but not proteins with more functional partners, exhibit low stochastic noise and low plasticity across environments. This presumably indicates the greater evolutionary constraints acting on intrinsically sticky proteins. Homo-oligomers also exhibit low noise and low plasticity, once their high level of stickiness is controlled for. This suggests that homo-oligomers might help mediate robustness to the consequences of noisy expression.

## Methods

### Protein abundance, TATA status and essentiality

Protein abundance measures were taken from Ghaemmaghami et al. [36] and subjected to a log transform. Classification of a gene’s promoter type as TATA(+) or TATA(−) was taken from Basehoar et al. [37]. The dispensability of each gene was identified using the essentiality classification of Mewes et al. [38].

### Noise

Noise values for 2168 genes were taken from Newman et al. [13], who used flow cytometry to measure the fluorescence of individual cells expressing GFP-fusion proteins from their endogenous promoters. The total coefficient of variance includes substantial contributions from variation in cell size and cell cycle state. We used the gated measurements of Newman et al. [13], which minimize the effects of these confounding factors. Newman et al. [13] reported their findings both as coefficients of variance (CV) and as a distance of each CV to a running median of CVs (referred to as DM). The DM values remove the strong and intrinsic effects of protein abundance on noise, and are the most appropriate for the study of evolutionary constraints. We therefore used the DM values, taken from cells grown in rich media. We performed an optimized Box-Cox transform (λ = −1.879) to make the data normal, as assessed by a Shapiro-Wilk test for normality. Note that noise data tended to be unavailable for genes expressed at low levels.

### Plasticity

mRNA expression data were downloaded from the Saccharomyces Genome Database [39,40]. We excluded 19 of the listed microarray papers on the grounds that they looked at conditions that wild yeast populations would not be expected to encounter, leaving 11 papers suitable for analysis [41-51].

Most papers included results from several trials. For example, Gasch et al. [44] contains expression data from cells using fructose, galactose, glucose, etc. as a carbon source. In contrast, Roberts et al. [41] contains expression data only from cells exposed to different concentrations and time durations of alpha factor. Our aim was to count one data point per biologically relevant environmental condition. In the first case, each microarray dataset was classified as its own experiment. In the second, since each microarray dataset involved alpha factor exposure, we grouped these as a single experiment. After we classified the microarrays within each paper, 27 independent experiments were obtained from the 11 papers.

**Figure 6:**
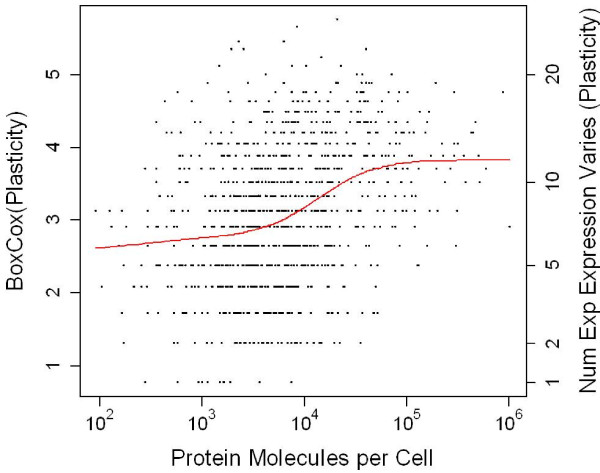
**Loess regression correcting plasticity for protein abundance.** Statistical analyses were performed on transformed plasticity numbers (left vertical axis), untransformed plasticity is shown right for illustration. Further analysis was performed on the deviate of each data point from the red loess regression line. The R loess regression function was used rather than the lowess function because loess returns residuals and better handles larger datasets.

If at least one measurement within an experiment showed a change in a gene’s expression by a factor of at least two relative to the experimental control, we counted that as an experiment for which that gene changed. For each gene, we counted the number of experiments in which a gene showed a change in expression at least as large as this arbitrary cut-off. We then performed an optimized Box-Cox transform (λ = 0.303) on this number, followed by a loess regression against protein abundance (Figure 6). Subsequent analyses were performed on the residuals from this loess regression, which are normally distributed as assessed by a Shapiro-Wilk test.

### Protein-protein interaction data, including self-interaction

The Y2H data were isolated from downloads of the BioGRID Interaction Database, Database for Interacting Proteins (DIP), IntAct database, and Molecular INTeraction Database (MINT) [52-59], yielding 29096 unique interactions from 1680 publications. No quality filter was applied to the Y2H data. The ACMS data were taken solely from the BioGRID Interaction Database [52,53], as BioGRID provides a comprehensive listing of data for that experiment type. To help reduce the influence of false positives in the ACMS data, we only kept interactions that appeared across 2 or more independent experiments: this is similar to the ACMS filtering procedure performed by Heo et al. [24]. Note that this further biased the ACMS data towards greater accuracy for more abundant proteins. After filtering, the “True” PPI consisted of 16786 unique interactions from 436 ACMS publications. Both Y2H and ACMS data were subjected to log transforms, yielding truncated normal data distributions, as assessed visually.

Self interaction status was assessed using Y2H data. Y2H data frequently contain false positive PPIs between proteins that would never be expressed in the same place and time. This drawback is clearly not a problem for the assessment of self-interactions, and Y2H rather than ACMS data were used to minimize false negatives.

### Regression models

**Figure 7:**
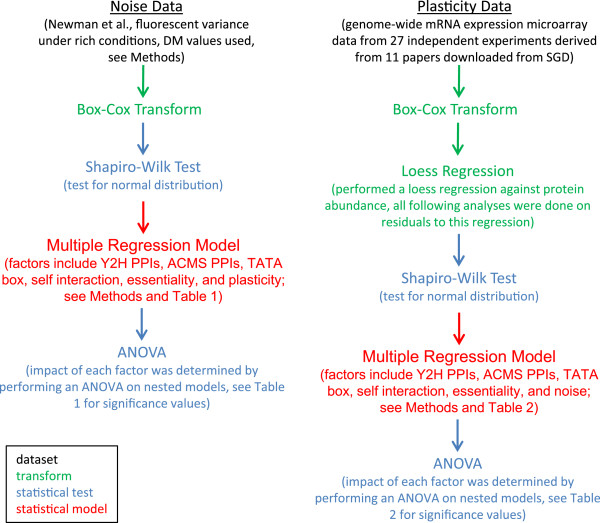
**Methods flowchart.** Simple illustrative flowchart showing progression of research methods including datasets analysed, data transforms, statistical tests, and regression models.

Multiple regression models were calculated using a linear regression function (lm) in the R statistical computing environment. Continuous variables (i.e. noise, plasticity, Y2H degree, and ACMS degree) were transformed, as described above, to make the data normal or nearly so and, in the case of noise and plasticity, to control for the confounding factor of protein abundance. Note, however, that both of the log-transformed PPI variables have truncated normal distributions, due to a floor at zero. The coefficients of determination of nested models were compared using an ANOVA. See Figure 7 for a flowchart demonstrating the research procedure.

## Competing interests

The authors declare that they have no competing interests.

## Authors' contributions

JM conceived the study. LB and JM contributed to the design of the study, to the analysis and interpretation of the results, and to writing the manuscript. LB carried out the study. All authors read and approved the final manuscript.
